# ‘Knowledge for better health’ revisited – the increasing significance of health research systems: a review by departing Editors-in-Chief

**DOI:** 10.1186/s12961-017-0248-y

**Published:** 2017-10-02

**Authors:** Stephen R. Hanney, Miguel A. González-Block

**Affiliations:** 1 Health Economics Research Group, Institute of Environment, Health and Societies, Brunel University London, Kingston Lane, Uxbridge, UB8 3PH United Kingdom; 2 Universidad Anáhuac, Av. Universidad Anáhuac 46, Lomas Anáhuac, 52786 Huixquilucan Mexico City, Mexico

**Keywords:** Agenda setting, Allocating funding, Capacity building, Equity, Health research systems, Health policies, Improved health, Knowledge production and translation, Research evaluation, Research impact assessment

## Abstract

How can nations organise research investments to obtain the best bundle of knowledge and the maximum level of improved health, spread as equitably as possible? This question was the central focus of a major initiative from WHO led by Prof Tikki Pang, which resulted in a range of developments, including the publication of a conceptual framework for national health research systems – Knowledge for better health – in 2003, and in the founding of the journal *Health Research Policy and Systems* (*HARPS*)*.*

As Editors-in-Chief of the journal since 2006, we mark our retirement by tracking both the progress of the journal and the development of national health research systems. *HARPS* has maintained its focus on a range of central themes that are key components of a national health research system in any country. These include building capacity to conduct and use health research, identifying appropriate priorities, securing funds and allocating them accountably, producing scientifically valid research outputs, promoting the use of research in polices and practice in order to improve health, and monitoring and evaluating the health research system. Some of the themes covered in *HARPS* are now receiving increased attention and, for example, with the assessment of research impact and development of knowledge translation platforms, the journal has covered their progress throughout that expansion of interest. In addition, there is increasing recognition of new imperatives, including the importance of promoting gender equality in health research if benefits are to be maximised. In this Editorial, we outline some of the diverse and developing perspectives considered within each theme, as well as considering how they are held together by the growing desire to build effective health research systems in all countries.

From 2003 until mid-June 2017, *HARPS* published 590 articles on the above and related themes, with authors being located in 76 countries. We present quantitative data tracing the journal’s growth and the increasing external recognition of its role. We thank the many colleagues who have kindly contributed to the journal’s success, and finish on an exciting note by welcoming the new Editors-in-Chief who will take *HARPS* forward.

## Editorial

“*He who receives an idea from me, receives instruction himself without lessening mine; as he who lights his taper* [candle] *at mine, receives light without darkening me. That ideas should freely spread from one to another over the globe, for the moral and mutual instruction of man, and improvement of his condition*” Thomas Jefferson, 1813 [1].

These inspiring words from a President of the United States of America were the basis for the use of a candle in the logo of *Health Research Policy and Systems* (*HARPS*). The candle is superimposed on a globe, thus reflecting the journal’s origins in WHO; Prof Tikki Pang founded the journal in 2003 during his tenure as Director of WHO’s then Research Policy and Cooperation Department. The growth of the journal reflects the expanding interest in conducting ‘research on research’ to study health research itself at diverse levels. The focus of such studies can include national research systems, international and local initiatives, and the various component parts that are increasingly recognised as being crucial for the success of research systems [2, 3]. Research on research can be conducted for a variety of purposes, including to strengthen the capacity to undertake scientifically valid and relevant research and to maximise and more equitably spread the benefits that can come from investing in research. It aims to do this by providing evidence to promote the effective use of the scare resources available for research, justify further research expenditure [4] and, in the case of the protocol by Greenhalgh et al. [2] published in August 2017, to conduct the research on research alongside a major investment in biomedical research in the United Kingdom in order to maximise the value of such investment.

After 11 years at the helm of *HARPS*, the departing Editors-in-Chief reflect herein on how the journal’s distinctive focus on building health research systems to improve health has evolved alongside the growing significance of the field itself. We then explore quantitative data tracing the journal’s growth, and the increasing external recognition of its role. We thank the many colleagues who have kindly contributed to the journal’s success and finish on an exciting note by welcoming the new Editors-in-Chief who will take the journal further forward.

Central themes maintained since the beginning of *HARPS* include research capacity building, agenda setting, the use of research outputs to improve healthcare, and the development and application of ways to assess such impact from health research. One of the major highlights occurred when the paper by Woelk et al. [5], ‘Translating research into policy: lessons learned from eclampsia treatment and malaria control in three southern African countries’, won the annual Medicine Award for the best paper in BioMed Central’s portfolio of medical research journals. Additionally, there has been increasing recognition of new imperatives, for example, of the importance of promoting gender equality in health research if benefits are to be maximised [6], and acknowledgement that evidence-based policymaking is not like evidence-based medicine [7]. Various of the themes covered in *HARPS* have been present for an astonishingly long time, some of which are now receiving increased attention (and, as with the assessment of research impact, *HARPS* has covered their progress throughout that expansion of interest), whereas other themes are newly emerging. We start by analysing what holds all these themes together before outlining some of the diverse and developing perspectives considered within each one.

### *HARPS*: a contribution to *‘*Knowledge for better health’

Tikki Pang established *HARPS* as part of the same WHO initiative that led to the article he and colleagues wrote, entitled ‘Knowledge for better health – a conceptual framework and foundation for health research systems’ [8]. A common impetus lay behind both, with the article stating: “*The central question is how to obtain the best ‘bundle’ of knowledge, and the resulting gains in health, out of the investments devoted to health research*” ([8], p. 817). The article identified a range of key components of a health research system for any country, including building capacity to conduct and use health research, identifying appropriate priorities, securing funds and allocating them accountably, producing scientifically valid research outputs, promoting the use of research in order to improve health, and monitoring and evaluating the health research system. These ideas were articulated in greater detail in WHO’s ‘World Report on Knowledge for Better Health’ [9], released at the Ministerial Summit on Health Research in Mexico in 2004, and followed by a World Health Assembly Resolution in 2005 committing its member states to strengthen their health research systems as the pathway to strengthening health systems [10]. We shall explore how *HARPS* has covered and promoted each of the topics set out above and thus made a growing contribution to ‘Knowledge for better health’.

#### Health research capacity building

Building the capacity to conduct and use research is often a challenge, especially in low- and middle-income countries (LMICs); *HARPS* has been a home for the reporting of accounts and analyses of this vital role. Many studies focus on health research capacity building in relation to one country or region, whereas others focus on particular fields or activities; for example, the very first article published in *HARPS* entitled ‘Assessing capacity for health policy and systems research in low and middle income countries’, by González-Block and Mills [11], was supported by the Alliance for Health Policy and Systems Research (AHPSR).

For some papers the focus on capacity building has been in a particular field of health research and for a specific region. A mini-series published in 2014 described how, in a programme funded by the United Kingdom’s Department for International Development (DFID) under the auspices of the Future Health Systems initiative, seven schools of public health and selected health policy institutions across six countries in East and Central Africa embarked on a 5-year project to strengthen their capacity to undertake high quality, policy-relevant health systems research [12]. Papers included one focused on experiences with applying a capacity assessment tool [13] and another on strengthening human and local financial resources for health systems research [14]. A separate DFID-funded study analysing capacity building schemes in health systems research focused on North–South partnerships between the London School of Hygiene and Tropical Medicine and institutions in South Africa and Thailand [15].

Other papers link the development of capacity to particular tasks. Three examples illustrate the continuing aspects of this theme. An early study in 2007 focused on capacity development for getting research into policy and practice in Laos [16]. In 2012, Bennett et al. [17] conducted a comparative study on approaches to developing the capacity of a health policy analysis institute in each of six countries (Bangladesh, Ghana, India, South Africa, Uganda and Vietnam). A recent study examined the building of capacity for information and communication technology use in global health research and training in China [18].

The development of networks and related skills can result from the positive impact arising from research capacity building training activities. Indeed, networking at the Canadian Coalition for Global Health Research annual Summer Institute for New Global Health Researchers contributed positively to the formation of a network of global health researchers [19]. A study of research training funded by the United States’ Fogarty International Centre in Uganda and Kenya [20] reported on the important contribution networking could make to tackling complex issues in health research such as the attainment of the Millennium Development Goals. Respondents interviewed in the evaluation confirmed that the programmes, “*consistently provided trainees with networking skills and exposure to research collaborations*” ([20], p. 1).

As noted, monitoring and evaluation are important within a health research system. Bates et al. [21], in one of several papers in *HARPS* from a Canadian–United Kingdom team, used peer-reviewed and grey literature to develop a five-step pathway for designing and evaluating health research capacity strengthening programmes, and tested it in a variety of contexts in Africa.

Finally, some of the capacity building articles in *HARPS* have contributed material that has been directly used for training purposes in both developed and developing countries. These include papers (described as guides) from the European Commission-funded SUPporting POlicy relevant Reviews and Trials (SUPPORT) Collaboration that were also translated into several languages. Not only have these SUPPORT papers been used in training courses, but some have been particularly widely accessed and/or cited, including the guide by Oxman et al. [22] introducing the concept of evidence-informed policymaking, and the one by Lavis et al. [23] analysing the role of policy briefs.

#### Research priority setting

The important health system function of research agenda setting has been covered by *HARPS* from diverse dimensions and often by papers that are both highly accessed and cited. These include one on the role of scoping studies in a United Kingdom programme of research into the organisation and delivery of health services [24], and a paper from WHO entitled ‘A checklist for health research priority setting: nine common themes of good practice’ [25]. Several national priority setting studies have drawn, in part at least, on the WHO checklist; for example, an analysis of previous attempts to set health research priorities in Panama [26], a commentary on health research priority setting in Brazil [27], and the development of a prioritised agenda for LMICs covering health policy and systems research (HPSR) on access to medicines [28]. Also worth mentioning in this context is a study on priorities for access to medicines research in Latin America and the Caribbean [29].

Considerable interest has been generated – as evidenced by the number of paper accesses and citations – by papers in *HARPS* on the continuing themes of the nature of agenda and priority setting for HPSR [30, 31], and by more recent papers which examined the nature of such research and called for it to become more people-centred [32]. Other papers have identified the priority areas for research into specific medical conditions, including a well-cited paper covering research and policy priorities in addressing birth asphyxia [33].

At the overall national level there is interest in how far research expenditure in particular fields matches the burden of disease within countries, for example, in Norway [34], and how national health research priorities have been identified, for example, in Timor-Leste through a scoping review of existing health data [35]. One of the major debates is the question of how far research priorities are best established internally by the scientific community themselves based on what they believe to be the most important unresolved issues, and how much of a key role other stakeholders should have. The long-running complex debate over this in the United Kingdom was skilfully analysed by Shergold and Grant [36]. If stakeholders other than researchers are to have a major role, which other groups should be involved and how? Other papers from this period included a review from Canada attempting to establish a collaborative research agenda for knowledge translation research in population health [37].

The theme of engaging more stakeholders in priority setting is gaining increasing attention in *HARPS*, including recently in a study funded by the United Kingdom’s Wellcome Trust of the role of non-governmental organisations (NGOs) in agenda setting in Malawi [38]. However, the engagement of patients and the public in priority setting is just one aspect of a wider move towards greater consumer involvement in health research, as various papers have analysed, including several from Australia [39, 40].

Some analyses of priority setting are part of wider studies of several aspects of health research systems. For example, a recent paper described the findings from a large-scale consultation exercise conducted by DFID to inform future global health research priorities as well as seeking views on research capacity and research uptake in developing countries [41], confirming the growing importance of non-communicable diseases as a global health research priority.

#### Securing funds and allocating them accountably

The above paper by Mc Conalogue et al. [41] was published in a collection, or thematic series, of papers funded in *HARPS* by WHO. The series, edited by Adam et al. [42] and entitled ‘Informing the Establishment of the WHO Global Observatory on Health Research and Development’, focuses in particular on the analysis of flows of research funding, which constitutes a key aspect of the work of the WHO Global Observatory. The securing of research funds and their allocation has long been a topic of interest to *HARPS*. In 2012, *HARPS* published a call by Terry et al. [43] for better mapping of how much the world is spending on health and disease-related research and development in order to facilitate attempts “*to align, or even begin to coordinate, health R&D investments with international public health priorities*” (p. 1). That paper, entitled ‘Mapping global health research investments, time for new thinking – A Babel Fish for research data’, echoed an earlier Commentary by one of the authors, Viergever [44], of how groups such as the Heads of International Research Organizations, which brings together major government and philanthropic funders of biomedical research, could help align financial flows for global health research towards public health priorities.

The important moves towards engaging more stakeholders in all aspects of health research systems has also involved experiments with ways of how they can best participate in making funding decisions. Here, papers include one from Australia on training consumers to review research [45] and others analysing a ‘virtual’ (computer-mediated) approach to health research commissioning in the United Kingdom [46], which was intended to enhance the accessibility, transparency and effectiveness of commissioning health research.

One of the major unresolved issues in the allocation of research funding is whether research is more productive if concentrated into a small number of centres or dispersed across many groups. This issue was addressed in a recent review in *HARPS* by Hernandez-Villafuerte et al. [47], concluding that the “*absence of predominant findings for or against the existence of economies of scale or scope implies a continuing need for case by case decisions when distributing research funding*” (p. 1).

#### Producing scientifically valid research outputs

The mapping of health research outputs by geography and/or topic, and analysis based on such mapping, can be of value to those funding and organising health research. Another early paper from González-Block [48], entitled ‘The state of international collaboration for health systems research: what do publications tell?’, set the pattern. A more recent paper by Rao et al. [49] reviewed the outputs of health systems research in India at a time when health system reform was being implemented in the country and made a series of important observations.

This theme is continued in the recent supplement ‘People and research: improved health systems for West Africans, by West Africans’ [50], funded by the Canadian International Development Research Centre and published in July 2017*.* One paper in this supplement analysed trends and patterns of peer-reviewed HPSR publications across the Economic Community of West African States (ECOWAS) [51]. One consideration was the degree of involvement of West African researchers in HPSR evidence generation in the sub-region, with the goal of using the findings “*to inform the development of a sub-regional strategy to strengthen HPSR and its use to inform development and improvement of health outcomes*” ([51], p. 1). In addition to the above supplement, we recently also started publishing a new collection entitled, ‘The State of Health Policy and Systems Research’, funded by the AHPSR where the increasing health policy and systems research capacity in LMICs is examined [52].

Papers focusing on analysis of the literature in specific disease areas include a review from the well-cited series on social science research and neglected tropical diseases [53], a bibliometric analysis of toxicology research productivity in Middle Eastern Arab countries between 2003 and 2012 [54], and a recently published 30-year bibliometric analysis of research coverage on HIV and AIDS in Lesotho [55].

#### Promoting the use of research in order to improve health

The use of research for societal benefit has a long history. As we outlined in a previous Editorial [3], almost four centuries ago Francis Bacon, one of the founders of the scientific method, described a utopian society in which some members of the scientific college would look at the experiments of their colleagues, “*and cast about how to draw out of them things of use and practice for man’s life and knowledge*” [56].

From its foundation, key themes of *HARPS* have been the exploration of how research evidence is transferred to policymakers and healthcare professionals in order to improve health (including how those processes might be enhanced), and the development of ways to assess the impact made by health research. The second paper published by *HARPS*, which we (and others) co-authored prior to becoming Editors, covered both these themes and analysed the use of research in health policies and how this should be assessed [57]. The importance of research transfer and its assessment is perhaps highlighted by the accesses (over 165,000) and citations (over 335 on Scopus) received by this paper.

Several major supplements in *HARPS* have examined the nature of the relationships between research and policy, including a supplement funded by DFID and led by the Liverpool School of Tropical Medicine on strengthening the research to policy and practice interface in sexual and reproductive health in resource poor contexts [58]. Similarly, a supplement from 2015 funded by DFID and the Australian Department of Foreign Affairs and Trade, and edited by Hirose et al. [59], entitled ‘Maternal and Newborn Health Research and Advocacy Fund, Pakistan’, described work aimed at bridging evidence, policy and practice to strengthen health systems for improved maternal and newborn health in Pakistan.

Diverse studies around the issues of research uptake have been reported in these supplements and other papers in *HARPS*. Some papers describe a specific case, for example, a study exploring the scaling up of research findings about translating the results from operations research into actions for expanding medical abortion services in rural health facilities in Nepal [60]. Other papers explore issues more widely, for example, the detailed analysis by Panisset et al. [61] focussed on the importance of implementation research evidence being taken up and used for policymaking to ensure progress towards achieving the Millennium Development Goals by LMICs. This paper discussed the use of implementation research by Knowledge Translation Platforms (KTPs), such as the WHO’s Evidence Informed Policy Network, and specifically analysed the scaling up of the use of zinc for the treatment of childhood diarrhoea in Bangladesh and of malaria treatment in Burkina Faso [61].

In 2006, *HARPS* published the series entitled ‘Improving the use of research evidence in guideline development’. This took a very broad perspective as reported in the Introduction by Oxman et al. [62], which explained how the WHO had asked its Advisory Committee on Health Research for advice on ways in which WHO could “*improve the use of research evidence in the development of recommendations, including guidelines and policies*” (p. 1). The Advisory Committee on Health Research established the Subcommittee on the Use of Research Evidence (SURE) to collect background documentation and to undertake a wide consultation across stakeholders. The SURE series was funded by WHO and the Norwegian Knowledge Centre for the Health Services and consisted of the various reviews of methods used in the development of guidelines. The most highly cited papers from the SURE series include the one by Schünemann et al. [63] on integrating values and consumer involvement into guideline development.

A more recent trio of papers in *HARPS* by Andermann et al. [64–66], entitled ‘Evidence for Health’, addressed a range of important issues in the better use of evidence for policy formulation, including the role of evidence in improving health and reducing inequities, overcoming critical barriers to the use of evidence and, in a final paper, ways of integrating values and context into evidence-informed decisions [66].

One-off papers also often include sophisticated analysis of the links between evidence and policymaking, as can be illustrated by three diverse examples. Pearson et al. [67] examined how an epistemic community informed policymaking on intentional self-poisoning in Sri Lanka. Second, Fraser et al. [68] explored the use of evidence by senior managerial decision-makers in the United Kingdom involved in the reconfiguration of stroke services in London. Third, Jansen et al. [69] analysed the dynamic process of developing an Academic Collaborative Centre for Public Health in the Netherlands with the objective of getting the three domains of policy, practice and research to become working partners.

The final example above links to an aspect of the use of research that is starting to emerge as another area receiving increased attention, namely, how far is it beneficial to have research-active healthcare staff? An Australian study published in 2017 identified the potential value of embedding Allied Health Profession research positions into healthcare settings [70]. Another study, also from 2017, reported on the findings of a retrospective study that suggested the research activity of the Oxford Biomedical Research Centre in the United Kingdom from 2007 to 2015 contributed to the effectiveness and efficiency of patient care at the local acute hospitals [71]. This partially echoes another theme in the WHO framework for health research systems, namely, that having research capacity in a country “*assists that country in learning, adapting, and benefiting from research conducted elsewhere*” ([8], p. 818).

#### Monitoring and evaluating the health research system, especially assessing impact

Papers about monitoring and evaluating various aspects of health research systems are important to *HARPS*. Some papers have already been noted, including ones assessing the impact of research use in policy [57] and evaluating capacity-building efforts [21]. Other papers described approaches developed to monitor the performance of major national research funders [72], and the importance of involving stakeholders in the development of a conceptual framework for an evaluation system for the global trials funded by the United States’ National Institute of Allergy and Infectious Diseases’ HIV/AIDS clinical trials network [73].

On the broad topic of research evaluation, *HARPS* focusses particularly on theoretical and empirical papers assessing the wider or societal impact of health research. The sixth paper published by *HARPS* was a major account of how the widely used Payback Framework for assessing health research impact is best applied [74]. As the topic of impact assessment has become one of growing importance globally, *HARPS* has played a major role in this, particularly by publishing various key reviews of studies assessing the impact of health research programmes, including reviews from the Italian Cochrane Centre [75] and other teams in Iran [76], Australia [77, 78] and the United Kingdom [79]. The latter review, published in 2017 in the WHO’s Global Observatory series [42], opens up another new area of research of considerable importance to health research systems, namely a focus on collating the findings of studies of the impact of health research in order to identify aspects of how the research programmes themselves are organised such that they may contribute to impacts being achieved. The review of assessments of the impact from 36 multi-project programmes described found very considerable diversity in the proportion of projects claiming to have made impacts such as informing health policy [79]. Several key factors were identified through the analysis of which aspects of programmes were linked to achieving the sometimes high levels of impact recorded. These factors included collaboration with potential users before and/or during the research, and conducting research to meet the needs of the healthcare system [79]. This paper also cited the statement in the World Health Report 2013 that “*adding to the impetus to do more research is a growing body of evidence on the returns on investment*” ([4], p. 46).

New approaches to impact assessment have been published in *HARPS*, including a list of indicators developed in France for assessing the outcome of translational cancer research [80], an approach for assessing research in low-resource settings in the Pacific Islands [81], two new frameworks from Australia that combined previous approaches [82, 83], and two new approaches from The Netherlands by Mostert et al. [84] and by Kok and Schuit [85]. The latter’s contribution mapping approach to assessing the impact of health research is increasingly informing other studies, including in The Netherlands [86], Ghana [87] and Canada [88].

There have also been historical papers on the nature of the impact of biomedical research in general in the United States [89] and of health services research in Mexico [90]. Empirical studies of the impact from the research of particular bodies of research include ones in Australia [91–95], Iran [96], Spain [97] and the United Kingdom [98]. Finally, a review of operations research in global health included a focus on the themes of health equity and impact [99].

We have also been able to make a greater contribution, especially to debates in innovative fields, following our recent decision to expand the categories of papers published in *HARPS*. This started in 2016 with the publication of selected Protocols provided they met rigorous standards, and continued in 2017 with the introduction of Opinion pieces. One example of this latter category was the paper by Cairney and Oliver [7] on evidence and policy noted earlier – it very quickly attracted a high level of attention on social media. Other interesting Opinion pieces have focused on assessing research impact in terms of the experience of transferring university-based research into the commercial sector in the United Kingdom [100], and an analysis from a Canadian team of experts as to whether research relevance – which is increasingly considered by research funders – is a necessary condition or stage in achieving impact, or a distinct aim of the research enterprise [101].

Consideration of several components of the research system had also been a feature of an early and highly accessed paper by Delisle et al. [102] for the Canadian Society for International Health. It focused on the roles of NGOs in health research systems, particularly in relation to global health research. It reported that “*NGOs are contributing at all stages of the research cycle, fostering the relevance and effectiveness of the research, priority setting, and knowledge translation to action.…Their contribution to more equitable, ethical, relevant and effective research is crucial and needs to be strengthened. Research has to be regarded as a broad loop system*” ([102], p. 1, 18–9).

Taking such a broad perspective is increasingly seen to be important. *HARPS* has recently published the latest research on developing ways to measure the often long periods between the start of biomedical research and its eventual uptake in policy and practice [103]. It is increasingly recognised that impacts from health research are more likely to occur when health research takes place in a system geared up to using the research to improve healthcare [8]. This leads neatly to the final, but crucial, theme we shall consider – the functioning of health research systems.

#### National health research systems

The importance of considering national health research systems is highlighted by an article in the new supplement published in July 2017, ‘People and research: improved health systems for West Africans, by West Africans’ [50]. The article by Sombié et al. [104] drew on the WHO framework for health research systems set out at the start of this editorial, plus earlier work from the Commission (later Council) on Health Research for Development (COHRED) [105], to evaluate a regional project that used a participatory approach to strengthen national health research systems in four post-conflict West African countries – Guinea-Bissau, Liberia, Sierra Leone and Mali.

This study noted there had been a range of earlier studies of health research systems in African countries, including several published in *HARPS*. One examined the current status and way forward for national health research systems in the WHO African Region as a whole [106]. Others took a national perspective, including an examination of the emergence and performance of a health research system in Guinea-Bissau [107], a Gambian perspective on the political undertones of building national health research systems [108], and an analysis of participatory approaches to legislative, institutional and networking dimensions of developing a national health research system in Zambia [109], which again drew on the earlier work of COHRED [105] and WHO [8]. There was also an earlier study by Sombié et al. [110] that explored the state of the research for health environment in the ministries of health of ECOWAS to “*provide relevant information about what aspects of national health research systems needs strengthening, so that research output can be relevant to meet national priorities for decision-making*” (p. 1). Exploration of the acute issues surrounding the research, and research system, needs in post-conflict and fragile states is becoming another sadly necessary theme in *HARPS*, with a further paper by Woodward et al. [111] also having explored the challenges of conducting health systems research in these circumstances.

As noted in our editorial in 2014 [3], we believe it is extremely useful to adopt a systems approach when considering how best to develop health research in all countries. We were therefore pleased that such an approach was promoted by the 2013 World Health Report [4]. The three key messages from the report included the statements, “*Research* [provides] *answers to improve human health…. All nations should be producers of research as well as consumers… To make the best use of limited resources, systems are needed to develop national research agendas, to raise funds, to strengthen research capacity, and to make appropriate and effective use of research findings*” ([4], p. xi). The report cited various papers from *HARPS* and explicitly stated, “*Research is likely to be most productive when it is conducted within a supportive national research system*” ([4], p. xv).

The importance of *HARPS* to the developing analysis of national health research systems is illustrated by the growing range of articles the journal has published on the topic. In addition to the papers described above, multi-country analyses of progress in developing health research systems include studies about the Latin American and Caribbean countries [112], the 27 countries of the European Union [113], and three African countries, namely Mozambique, Senegal and Tanzania [114]. Country-specific papers include those on Panama [115], the Solomon Islands [116] and England [117]. The latter paper is one of many that highlight both the complexity, but also the appropriateness, of taking a systems approach to health research at the national level in order to enhance the opportunities for benefitting to the maximum extent from the investments made in health research. This will remain a key theme for *HARPS*.

### Some facts and figures and thanks

The broad range of themes developed by *HARPS* from 2003 up until mid-June 2017 were published in a total of 590 articles, of which 67% were Research articles and the rest mostly Reviews and Commentaries, although Editorials, and more recently Protocols and Opinions, also figure, with some series also featuring Introductions and Guides. A total of 10 series were published through prior arrangement with diverse institutions, some of which have been noted above.

**Fig. 1:**
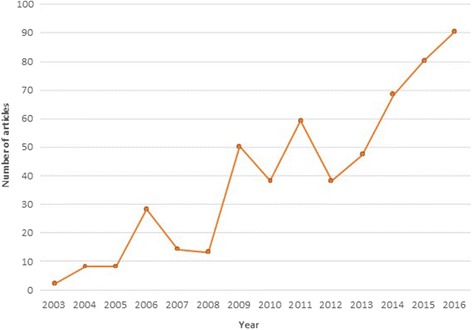
Number of articles published by *Health Research Policy and Systems* since its foundation in 2003 up until 2016

Publications showed a steady increase through the years (Fig. 1). In its first year, only two papers were published, climbing to eight in 2004 and 2005. An important increase was observed in 2006, with 28 papers, demonstrating the journal’s viability. Publications rose significantly in 2009, more than doubling from previous years. Between 2009 and 2013 publications fluctuated around an average of about 45 articles per year. In the summer of 2013 the journal obtained its official Impact Factor by Thomson Reuters’ Journal Citation Reports, leading to a subsequent steady increase in the number of publications. The impact factor has increased each year, reaching 2.3 in the June 2017 announcement of the figure for 2016. Today, *HARPS* is fully consolidated as a niche journal within a range of wider fields, including HPSR, science studies and research on research. In 2016, a total of 90 papers were published, while for 2017, *HARPS* is on track to publish approximately 100 papers.

Authors publishing in *HARPS* come from a wide range of institutions, with at least 882 distinct units at the department, division or programme level. Academic and research institutions have predominated, followed by development agencies. A total of 76 countries have been represented, of which 30 are high-income countries, 16 upper middle-income countries, 19 lower middle-income countries and only 11 low-income countries. High-income country authors (or members of institutions in high-income countries, which often include authors originally from LMICs) are the most represented, with 71.4% of the total authorships between 2012 and 2017, followed by lower middle-income (11.8%), upper middle-income (10.7%) and low-income country authors (6%). The countries most represented in authorships are the United Kingdom (14.7% of the total), followed by Australia (9.8%) and the United Sates (9.1%).

In conclusion, *HARPS* has shown a steady progress across the years, with an increasing number of research articles and special series, and a focus on an increasing range of topics of growing significance. Publication of the first, WHO-linked, series in 2006 plus indexing by Thomson Scientific were major milestones. However, there is a need to promote research on health research systems among LMIC authors and institutions, as well as more broadly across high-income country institutions.

We owe a debt of gratitude to many people without whom such progress of the journal would not have been possible. We would like to thank the hundreds of colleagues who have submitted articles and those who have most kindly freely given their time to provide expert peer-review. We were fortunate to have inherited the Editorial Board of leading experts that Tikki Pang had assembled; we are most grateful for their input, and that of further colleagues who we have recruited. We have received strong support from the team at BioMed Central, and have especially appreciated the enthusiastic encouragement and expert advice we have received from Dr Liz Hoffman, our Journal Development Officer, for many years. Finally, above all, the Editors are grateful for the outstanding and highly skilled support we have received from Dr Rosanna Gonzalez-McQuire, who has been the journal’s Managing Editor since 2008 and without whose almost daily input the journal would not have had the success that it has.

### Welcome to the future

We are delighted to announce the new Editors-in-Chief of *HARPS*, two highly talented academics from among the next generation of leaders of the research on research field. They are Dr Tari Turner, Senior Research Fellow at Cochrane Australia based at Monash University, Melbourne, and Prof Fadi El-Jardali, Co-Director of the WHO Collaborating Center for Evidence-Informed Policy and Practice, based at the American University of Beirut, Lebanon. Both have a long history of publishing in *HARPS*, and elsewhere, on the use of research and evidence in health policymaking, and other topics as well.

Tari’s first papers in *HARPS* described the results of the wonderfully named South East Asia Optimising Reproductive and Child Health in Developing Countries (SEA-ORCHID) project of which she was part [118, 119]. It was a 5-year collaborative project between Thailand, Malaysia, the Philippines, Indonesia and Australia funded by the Wellcome Trust and the Australian National Health and Medical Research Council. The aims of SEA-ORCHID were to address whether the health of mothers and babies in the four South East Asian countries could be improved “*by increasing the capacity for research synthesis and improving the implementation of effective interventions*” ([118], p. 2). Among her other more recent projects, Tari has been working on collaborations between Monash University, the Sax Institute in Sydney and others, aimed at increasing the use of health research, for example, through development and validation of SEER (Seeking, Engaging with and Evaluating Research), a measure of policymakers’ capacity to engage with and use research [120]. It was thought to be a necessary adjunct to strategies for increasing research use because it addressed the lack of well-validated measures for policy contexts which had “*hampered efforts to identify priorities for capacity building and to evaluate the impact of strategies*” ([120], p. 1).

Fadi first started publishing in *HARPS* in 2011 and was lead author of a study that provided the first stocktaking of HPSR published and conducted in the Eastern Mediterranean Region [121]. It examined the output in 12 countries, identifying gaps and assessing “*the extent to which existing HPSR produced in the region addresses regional priorities*” ([121], p. 1). Fadi is Professor of Health Policy and Systems at the American University of Beirut and also the Director of its Knowledge to Policy (K2P) Center. In addition, he is an adjunct professor at the Department of Health Research Methods, Evidence, and Impact at McMaster University in Canada. Through his work to identify ways to promote knowledge translation, he was part of the KTPs Evaluation Team that conducted a print media analysis in 44 countries in Africa, the Americas, Asia and the Eastern Mediterranean in order to increase understanding of the climate for evidence-informed health systems and to provide a baseline for an evaluation of KTPs [122]. A later paper described a multi-method analysis of KTPs that are being established in LMICs to enhance evidence-informed health policymaking, and concluded that they were “*a promising development in supporting EIHP* [evidence-informed health policymaking] *… Lessons learned can help to promote similar EIHP initiatives in other countries*” ([123], p. 1).

Given their clear focus on a range of issues of central importance to *HARPS*, we warmly welcome Tari Turner and Fadi El-Jardali.

## Abbreviations

AHPSR: Alliance for Health Policy and Systems Research
COHRED: Council on Health Research for Development
DFID: Department for International Development
ECOWAS: Economic Community of West African States
HARPS: Health Research Policy and Systems
HPSR: health policy and systems research
KTPs: Knowledge Translation Platforms
LMICs: low-and-middle-income countries
NGOs: Non-Governmental Organisations
SEA-ORCHID: The South East Asia Optimising Reproductive and Child Health in Developing Countries (SEA-ORCHID) project
SUPPORT: SUPporting POlicy relevant Reviews and Trials (SUPPORT) Collaboration
SURE: Subcommittee on the Use of Research Evidence.
